# Reproductive factors and cardiometabolic disease among middle-aged and older women: a nationwide study from CHARLS

**DOI:** 10.3389/fcvm.2024.1345186

**Published:** 2024-04-30

**Authors:** Qiong Wang, Bo Pang, Jing Wu, Chunyan Li, Wenquan Niu

**Affiliations:** ^1^ Graduate School, Beijing University of Chinese Medicine, Beijing, China; ^2^ Department of Pediatrics, China-Japan Friendship Hospital, Beijing, China; ^3^ Center for Evidence-Based Medicine, Capital Institute of Pediatrics, Beijing, China; ^4^ Department of Cardiology, Integrated Traditional Chinese and Western Medicine, China-Japan Friendship Hospital, Beijing, China

**Keywords:** China Health and Retirement Longitudinal Study, coronary heart disease, computer-assisted personal interviewing, reproductive factors, cardiometabolic disease, CHARLS, risk factor

## Abstract

**Background:**

Cardiometabolic disease is skyrocketing to epidemic proportions due to the high prevalence of its components and the aging of the worldwide population. More efforts are needed to improve cardiometabolic health. The aim of this nationally representative study based on the China Health and Retirement Longitudinal Study (CHARLS, 2014–2018) was to examine the association between reproductive factors and cardiometabolic disease among Chinese women aged ≥45 years.

**Methods:**

The CHARLS is an ongoing longitudinal study initiated in 2011, and the latest follow-up was completed in 2018. In total, 6,407 participants were analyzed. Effect-sizes are expressed as odds ratios (OR) and 95% confidence intervals (CI). Confounding was considered from statistical adjustment, subsidiary exploration, and unmeasured confounding assessment aspects.

**Results:**

Of 6,407 accessible participants, 60.9% were recorded as having one or more of five predefined cardiovascular or metabolic disorders. Compared to those with two children, participants who had 0–1 child were found to have a lower risk of cardiometabolic disease (OR = 0.844, 95% CI: 0.714–0.998), and those who had ≥3 children had a greater risk (OR = 1.181, 95% CI: 1.027–1.357). Age at menarche of 16–18 years was a protective factor compared with ≤16 years of age (OR = 0.858, 95% CI: 0.749–0.982). In contrast, participants with a history of abortion were 1.212 times more likely to have cardiometabolic disorders (OR = 1.212, 95% CI: 1.006–1.465). The likelihood for the presence of unmeasured confounding was low, as reflected by E-values.

**Conclusions:**

Our findings demonstrate that number of children, age at menarche, and history of abortion were associated with a significant risk of cardiometabolic disease among Chinese women aged ≥45 years.

## Introduction

Cardiometabolic disease, covering a broad group of cardiovascular and metabolic disorders with shared pathological foundations, has caused an enormous healthcare burden that is expected to rise due to the aging of the worldwide population (1, 2). The Global Burden of Disease 2019 study estimates that approximately 20% and 10% of deaths are attributable to hypertension and diabetes mellitus, respectively (3). In China, an estimated 330 million individuals are affected by cardiovascular disease, which was responsible for approximately two in five deaths in 2021 (4). It is hoped that more endeavors will curb this global burden by identifying high-risk individuals and developing preventive strategies to improve cardiometabolic health.

Evidence is accruing favoring the contributory roles played by reproductive factors in the onset and progression of cardiometabolic disease, especially among middle-aged and older women. For instance, increasing numbers of children was widely recognized as a promising risk factor for coronary heart disease (CHD) and mortality of mothers (5–8), and it also exerted an unfavorable impact on longevity (9). In support of this claim, two studies among middle-aged and older Chinese women have reported that increasing numbers of children can remarkably increase the risk of multimorbidity and the number of chronic conditions (8, 10). However, in an early British birth cohort, number of children showed no consistent relationship with CHD risk profiles at the age of 53 years (11). As for age at menarche and menopause (12, 13), the same was true, with no consensus on their implications, mainly due to the ethnic diversity of study participants, limited size of most observational studies, and insufficient consideration of residual confounding. Hence, the topic on the association between reproductive factors and cardiometabolic disease is still subject to an ongoing debate and requires further exploration.

To shed more light on this topic and provide an evidence base for future investigations, we aimed to test the hypothesis that reproductive factors were strongly associated with cardiometabolic disease, both as a whole and individually, among women aged ≥45 years by analyzing the longitudinal data from the China Health and Retirement Longitudinal Study (CHARLS, 2014–2018).

## Methods

### Study participants

The present study used data from the CHARLS, which is an ongoing, large-scale, multistage, nationally representative health survey. Briefly, 17,708 participants in 10,257 households, 450 villages, and 150 counties and districts from 28 provinces were enrolled between 2011 and 2012 (wave I). After the baseline interview, follow-up surveys were performed in 2013 (wave II), 2015 (wave III), and 2018 (wave IV). In 2014, a new life course survey was conducted to construct the life history of Chinese residents by tracing past experiences of the participants.

**Figure 1:**
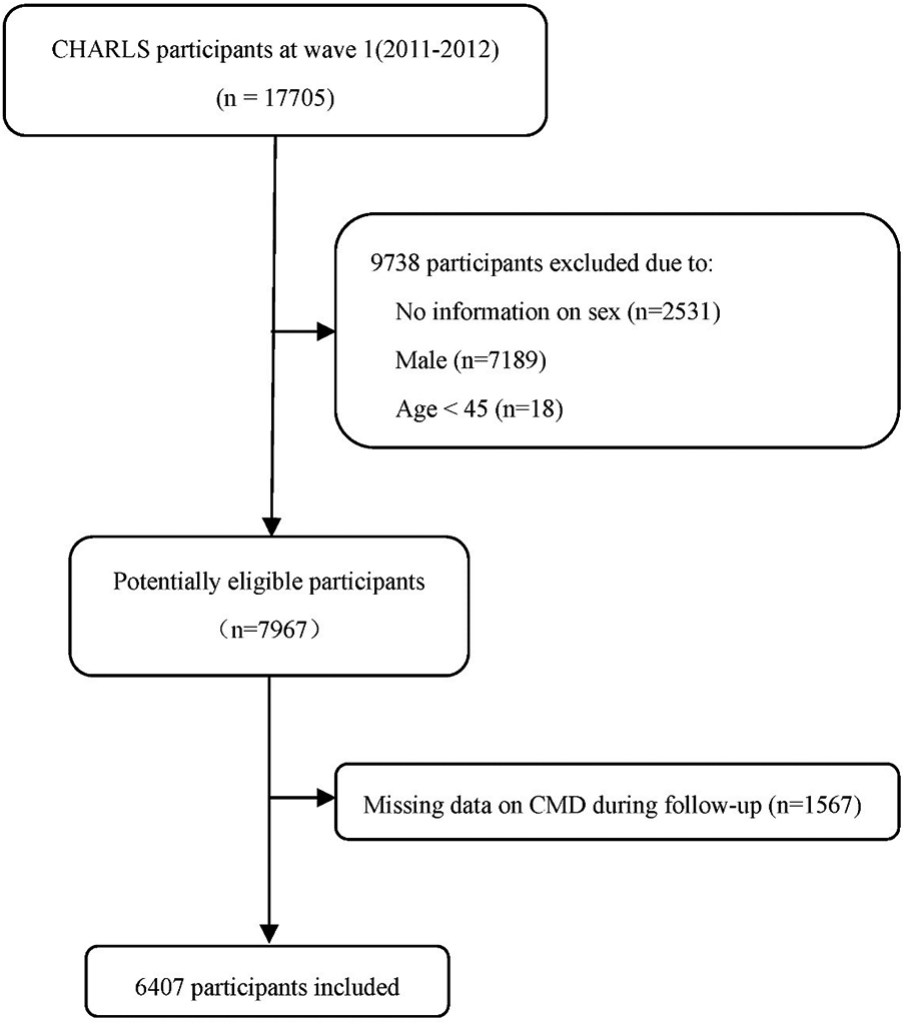
Selection flowchart of 6,407 eligible participants in this study.

In this study, participants in the CHARLS life history survey (2014) and the latest follow-up (wave IV) were abstracted, and only women aged ≥45 years were analyzed (*n* = 7967). After excluding 1,560 participants with missing information on any of five common cardiometabolic disease, including hypertension, dyslipidemia, diabetes mellitus, heart problems (heart attack, coronary heart disease, angina, or congestive heart failure), and stroke, 6,407 participants remained in the final analysis. The participant flow chart is shown in Figure 1.

CHARLS surveys were conducted following stringent quality control policies. For each round of surveys, face-to-face interviews were conducted by trained interviewers via computer-assisted personal interviewing (CAPI). Information on demographics, lifestyles, dietary intake, physical activity, and medical history was recorded in well-designed structured questionnaires. More details about CHARLS surveys can be found elsewhere (14).

The study protocol was reviewed and approved by the Institutional Review Board of Peking University (IRB00001052-11015). Written informed consent was obtained from all participants.

### Cardiometabolic disease

In this study, the five cardiovascular and metabolic disorders mentioned above are collectively defined as cardiometabolic disease. The five disorders were determined by the following questions in the CHARLS surveys: “Have you been diagnosed with hypertension by a doctor?”, “Have you been diagnosed with dyslipidemia by a doctor?”, “Have you been diagnosed with diabetes by a doctor?”, “Have you been diagnosed with heart problems by a doctor?”, and “Have you been diagnosed with stroke by a doctor?” The presence of any cardiometabolic disease under investigation was recorded if the answer to any of the five questions above was “yes,” as defined previously (15).

### Reproductive factors

Information on number of children was abstracted from the 2014 CHARLS life history survey and was defined based on the rank of biological children. Age at menarche and history of abortion were gleaned from wave I to wave IV, whichever was recorded first. Menopausal status and age at menopause were extracted from wave IV. Age at first live birth was calculated by biological age and age of the oldest child. Duration of reproductive period was calculated as the period from age at menarche to age at menopause.

### Statistical analyses

The characteristics of participants by cardiometabolic disease are presented by *n* (%) for categorical variables and mean (SD) or median [interquartile range (IQR)] for continuous variable. The distributions of variables were compared using the independent *t*-test, Mann–Whitney U test, χ2 test, or Fisher's exact test, where appropriate. To examine the association between reproductive factors and cardiometabolic disease, multivariable logistic regression models were undertaken to estimate odds ratios (OR) and 95% confidence intervals (CI). Model 1 was unadjusted. Age (continuous, years), residential area (rural, urban), and education (illiterate, literate) were adjusted in Model 2, and body mass index (BMI; <24, 24–28, ≥28 kg/m^2^) (16) was additionally adjusted in Model 3. The E-value was adopted to assess the potential impact of unmeasured confounding, with the higher E-values corresponding to the stronger the unmeasured confounders needed to explain observed association (17, 18). In addition, stratified analyses were conducted according to age (≤65 and >65 years), residential area (rural, urban), education (illiterate, literate), and BMI (<24, 24–28, ≥28 kg/m^2^), respectively. Effect-size estimates are expressed as OR and 95% CI.

Further dose-response associations between reproductive factors and cardiometabolic disease were examined using restricted cubic spline analyses. In addition, the selection of optimal cutoff point for age associated with cardiometabolic disease was explored using restricted cubic spline analyses, with the most appropriate knot number determined by the Akaike Information Criterion (AIC). The differential prediction probabilities of reproductive factors were illustrated upon stratification by age, residential area, education, and BMI, respectively.

All analyses were performed using STATA software (version 14.0, Stata Corp., College Station, TX, USA) and R (version 4.3.1, R Foundation, Vienna, Austria). A two-sided *p*-value <0.05 was considered statistically significant.

## Results

### Characteristics of study participants

The characteristics of the 6,407 study participants in wave IV according to cardiometabolic disease are shown in Table 1. The median age was 57.8 years (IQR 8.8), and 3,900 (60.9%) participants were recorded as having at least one cardiometabolic disease. Compared with those without cardiometabolic disease, participants with cardiometabolic disease were older and more likely to be urban residents and also had a lower level of education and a higher BMI.

**Table 1:** Characteristics of 6,407 study participants according to cardiometabolic disease status in this study.

Characteristics	Subgroups	Overall	Cardiometabolic diseases		*p*
		(*n* = 6,407)	Absence (*n* = 2,507)	Presence (*n* = 3,900)	
Age (years)		65.0 [58.0–73.0]	63.00 [56.0–69.0]	67.00 [60.0–74.0]	<0.001
Age group	≤65 years	3,311 (51.7)	1,583 (63.2)	1,728 (44.3)	<0.001
	>65 years	3,090 (48.3)	920 (36.8)	2,170 (55.7)	
Residential area	Rural	5,301 (82.8)	2,170 (86.7)	3,131 (80.3)	<0.001
	Urban	1,099 (17.2)	332 (13.3)	767 (19.7)	
Education	Illiterate	2,573 (40.2)	990 (39.6)	1,583 (40.6)	0.40
	Literate	3,822 (59.8)	1,509 (60.4)	2,313 (59.4)	
Body mass index	Not overweight/obesity	2,798 (52.8)	1,371 (65.4)	1,427 (44.5)	<0.001
	Overweight	1,719 (32.4)	586 (27.9)	1,133 (35.4)	
	Obesity	786 (14.8)	141 (6.7)	645 (20.1)	
Age at menarche		16.00 [15.0–18.0]	16.00 [15.0–18.0]	16.00 [15.0–18.0]	0.90
Age at menopause		45.00 [2.0–50.0]	45.00 [1.3–50.0]	45.00 [2.0–50.0]	0.83
Age at first livebirth		27.00 [24.0–30.0]	27.00 [24.0–30.0]	27.00 [24.0–30.0]	0.20
Duration of reproductive period		26.00 [14.0–35.0]	26.00 [15.0–35.0]	26.00 [14.0–35.0]	0.60
History of abortion	No	5,221 (88.3)	2,070 (89.3)	3,151 (87.6)	0.06
	Yes	692 (11.7)	248 (10.7)	444 (12.4)	

Data are expressed as median [interquartile range] for skewed continuous variables or mean (standard deviation) for normally distributed continuous variables or number (%) for categorical variables. The *p*-value was calculated by *χ*^2^ test or Fisher’s exact test for categorical variables, by independent *t-*test for normally distributed continuous variables, and by Mann–Whitney test for skewed continuous variables.

### Association of reproductive factors with cardiometabolic disease

The association between six reproductive factors and cardiometabolic disease is presented in Table 2. Compared to participants with two children, the risk for cardiometabolic disease was significantly reduced by 15.6% in participants with 0–1 child but increased by 18.1% in participants with ≥3 children. In addition, age at onset of menarche between 16 and 18 years was a protective factor compared with ≤16 years of age. Women with a history of abortion were found to have a greater association with cardiometabolic disorders. No significant association was seen for age at first live birth, age at menopause, and duration of reproductive period.

**Table 2:** Overall association of fertility and reproductivity-related factors with the risk for cardiometabolic diseases before and after adjusting for confounding factors among 6,407 eligible participants.

Variables	Model 1	Model 2	Model 3
Number of children			
0–1	1.366 (1.221–1.529)**	0.903 (0.779–1.045)	0.844 (0.714–0.998)*
2	Ref.	Ref.	Ref.
≥3	1.827 (1.699–1.964)**	1.127 (0.993–1.279)	1.181 (1.027–1.357)*
Age at first livebirth (years)			
<25	1.338 (1.122–1.597)**	0.962 (0.782–1.183)	0.867 (0.688–1.093)
25–34	Ref.	Ref.	Ref.
≥34	1.464 (1.110–1.932)**	0.941 (0.692–1.280)	0.948 (0.681–1.319)
Age at menarche (years)			
≤16	Ref.	Ref.	Ref.
16–18	1.551 (1.418–1.697)**	0.870 (0.767–0.988)*	0.858 (0.749–0.982)*
>18	1.583 (1.430–1.754)**	0.852 (0.742–0.977)*	0.867 (0.748–1.004)
Age at menopause (years)			
≤45	0.994 (0.796–1.241)	1.062 (0.766–1.473)	0.864 (0.580–1.287)
45–55	Ref.	Ref.	Ref.
≥55	1.211 (0.659–2.223)	1.097 (0.560–2.146)	0.881 (0.365–2.124)
Duration of reproductive period (years)			
≤33	1.054 (0.841–1.321)	1.047 (0.732–1.499)	0.995 (0.663–1.492)
33–43	1.00	1.00	1.00
≥43	NA	NA	NA
History of abortion			
No	Ref.	Ref.	Ref.
Yes	1.762 (1.510–2.056)**	1.169 (0.989–1.385)	1.212 (1.006–1.465)*

Ref., reference group; NA, data not available.

Data are expressed as odds ratio (95% confidence interval). Model 1: no confounder was adjusted. Model 2: age (continuous), residential area (rural, urban), and education (illiterate, literate) were adjusted. Model 3: age (continuous), residential area (rural, urban), education (illiterate, literate), and body mass index were adjusted.

* *p *< 0.05.

** *p *< 0.01.

In addition, the association of six reproductive factors with its five components was also examined (Supplementary Tables 1–5).

### Dose-response association

**Figure 2:**
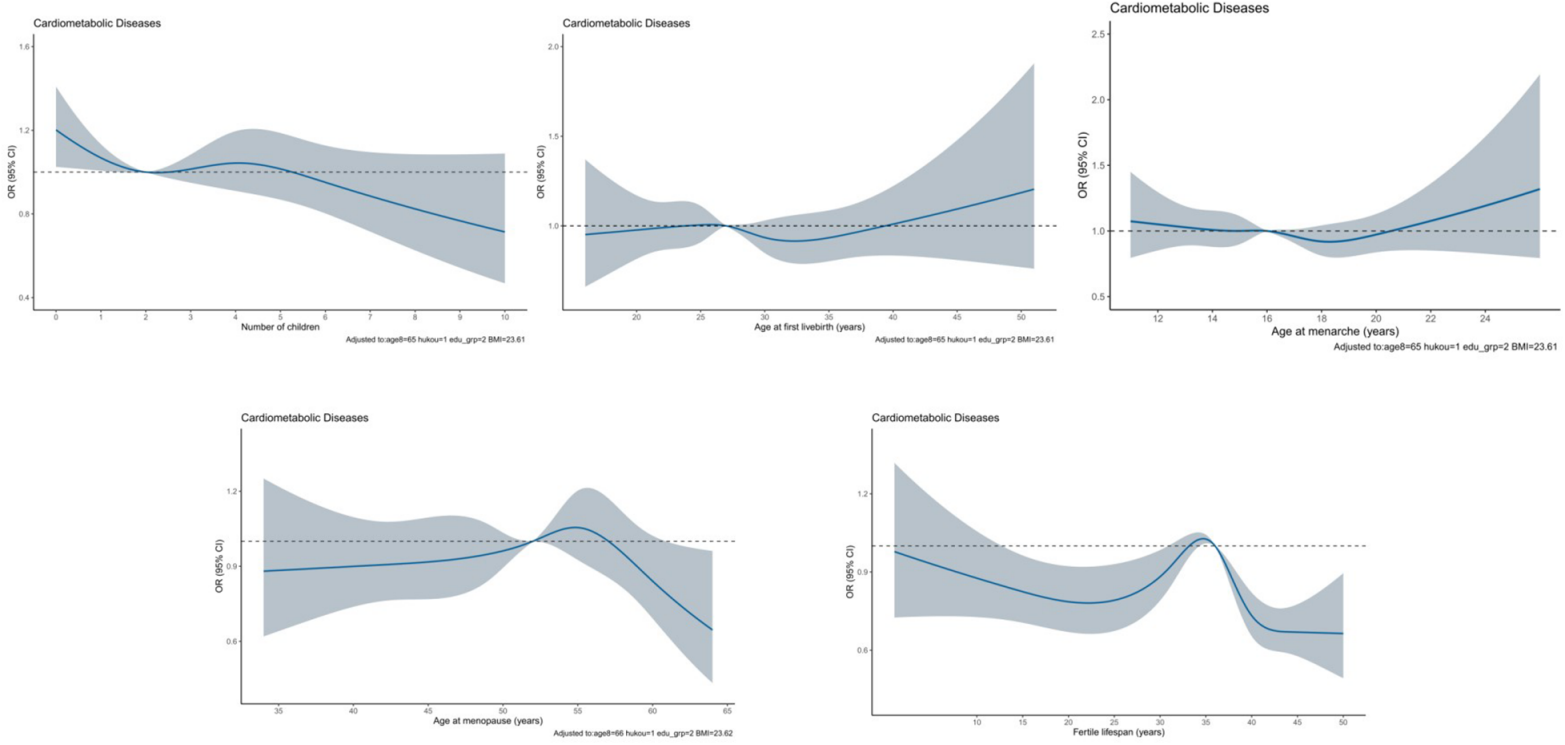
Dose-response association between reproductive factors and cardiometabolic disease by using restricted cubic spline method among 6,407 eligible participants. The solid blue line represents the OR for cardiometabolic disease with the increasing number of children. The gray shadow represents the 95% CI of OR. OR and 95% CI were calculated after adjusting for age, residential area, education, and body mass index.

Figure 2 illustrates the dose-response association with cardiometabolic disease for five continuous reproductive factors. Visual inspection revealed the non-linear association of number of children, age at menopause, and duration of reproductive period with cardiometabolic disease, as confirmed by statistical tests (*p* < 0.01).

### Stratified analyses

**Figure 3:**
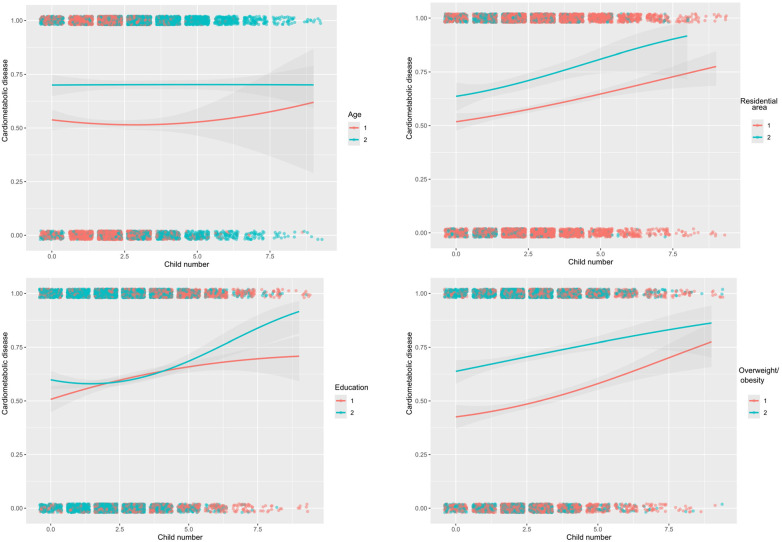
Estimated probabilities of cardiometabolic disease with the increasing number of children by age (≤65 and >65 years), residential area (rural, urban), education (illiterate, literate), and overweight/obesity (non-overweight/obesity, overweight/obesity) among 6,407 eligible participants.

Subsidiary analyses were undertaken according to confounding factors when assessing the association between reproductive factors and cardiometabolic disease (Table 3 and Figure 3). Small numbers of children tended to be associated with the reduced risk of cardiometabolic disease, especially in participants aged ≥65 years (OR = 0.702, 95% CI: 0.499–0.983), with urban household (OR = 0.672, 95% CI: 0.463–0.971), being literate (OR = 0.698, 95% CI: 0.573–0.850), and being overweight or obese (OR = 0.778, 95% CI: 0.609–0.993). By contrast, large numbers of children were also a protective factor for cardiometabolic disease in participants aged ≥65 years (OR = 0.751, 95% CI: 0.604–0.934), yet a risk-conferring factor in participants being illiterate (OR = 1.468, 95% CI: 1.171–1.839) and non-overweight (OR = 1.230, 95% CI: 1.018–1.456).

**Table 3:** Subsidiary associations of fertility and reproductivity-related factors with cardiometabolic disease among 6,407 eligible participants.

Reproductive factors	Age (years)		Residential area		Education		Overweight/Obesity	
	≤65	>65	Rural	Urban	Illiterate	Literate	No	Yes
	OR (95% CI)	OR (95% CI)	OR (95% CI)	OR (95% CI)	OR (95% CI)	OR (95% CI)	OR (95% CI)	OR (95% CI)
Number of children								
0–1	0.940 (0.770–1.147)	0.702 (0.499–0.983)*	0.832 (0.686–1.009)	0.672 (0.463–0.971)*	1.062 (0.767–1.474)	0.698 (0.573–0.850)**	0.922 (0.725–1.172)	0.778 (0.609–0.993)*
2	Ref.	Ref.	Ref.	Ref.	Ref.	Ref.	Ref.	Ref.
≥3	0.844 (0.705–1.001)	0.751 (0.604–0.934)*	1.21 (1.020–1.372)*	1.294 (0.818–1.979)	1.468 (1.171–1.839)**	1.051 (0.880–1.254)	1.230 (1.018–1.456)*	1.075 (0.869–1.330)
Age at first livebirth (years)								
<25	0.984 (0.753–1.288)	0.872 (0.523–1.376)	0.914 (0.715–1.169)	0.794 (0.375–1.736)	0.894 (0.594–1.348)	0.854 (0.645–1.132)	1.275 (0.919–1.768)	0.691 (0.498–0.957)*
25–34	Ref.	Ref.	Ref.	Ref.	Ref.	Ref.	Ref.	Ref.
≥34	0.872 (0.534–1.423)	0.637 (0.408–1.000)*	0.955 (0.676–1.354)	1.027 (0.324–3.906)	1.112 (0.666–1.874)	0.836 (0.541–1.297)	0.881 (0.559–1.390)	0.873 (0.523–1.458)
Age at menarche (years)								
≤16	Ref.	Ref.	Ref.	Ref.	Ref.	Ref.	Ref.	Ref.
16–18	0.783 (0.658–0.932)*	0.804 (0.647–1.00)*	0.857 (0.734–0.986)*	0.931 (0.652–1.330)	0.803 (0.641–1.005)	0.895 (0.755–1.060)	0.934 (0.775–1.125)	0.809 (0.659–0.993)*
>18	0.822 (0.673–1.004)	0.693 (0.560–0.857)**	0.858 (0.723–0.992)	1.15 (0.743–1.786)	0.848 (0.675–1.065)	0.909 (0.748–1.105)	1.003 (0.827–1.216)	0.792 (0.624–1.005)
Age at menopause (years)								
≤45	1.048 (0.688–1.599)	0.397 (0.109–1.450)	0.937 (0.605–1.451)	0.867 (0.284–2.643)	0.482 (0.196–1.185)	0.945 (0.608–1.470)	0.826 (0.465–1.468)	0.993 (0.568–1.734)
45–55	Ref.	Ref.	Ref.	Ref.	Ref.	Ref.	Ref.	Ref.
≥55	0.759 (0.248–2.326)	0.477 (0.102–2.223)	0.733 (0.270–1.994)	3.594 (0.304–42.498)	0.537 (0.137–2.103)	1.408 (0.440–4.504)	1.606 (0.143–6.244)	0.572 (0.178–1.835)
Fertile lifespan (years)								
<33	1.019 (0.62–1.567)	1.125 (0.306–4.134)	0.970 (0.617–1.524)	1.442 (0.510–4.074)	0.530 (0.213–1.317)	1.091 (0.696–1.710)	0.891 (0.486–1.633)	1.167 (0.662–2.057)
33–43	Ref.	Ref.	Ref.	Ref.	Ref.	Ref.	Ref.	Ref.
>43	NA	NA	NA	NA	NA	NA	NA	NA
History of abortion	1.118 (0.878–1.422)	1.550 (1.123–2.140)**	0.993 (0.804–1.229)	1.789 (1.177–2.719)**	1.076 (0.758–1.530)	1.178 (0.944–1.469)	1.363 (1.052–1.767)*	1.055 (0.797–1.397)

Ref., reference group; NA, data not available.

Subgroup analyses were conducted by four confounders (age, residential area, education, and overweight/obesity), and for each subgroup, the other three confounders were adjusted when calculating OR and 95% CI.

* *p *< 0.05.

** *p *< 0.01.

As for age at first live birth (Table 3), significance was merely seen in older participants who had their first live birth at 34 years or more (OR = 0.637, 95% CI: 0.408–1.000) and in overweight or obese participants who had their first live birth at younger than 25 years (OR = 0.691, 95% CI: 0.498–0.957).

Regarding age at menarche, there was a significantly reduced risk of cardiometabolic disease for menarche age striding 16–18 years, irrespective of age groups, as well as in participants living in rural areas and being overweight or obese. No significance was observed for age at menopause and duration of reproductive period across all subgroups.

Table 3 shows that having a history of abortion was found to have a greater risk of cardiometabolic disease in older participants (>65 years) (OR = 1.550, 95% CI: 1.123–2.140), as well as in participants living in urban areas (OR = 1.789, 95% CI: 1.177–2.719) and being non-overweight (OR = 1.363, 95% CI: 1.052–1.767).

Viewing the significant contribution of number of children, the probabilities of cardiometabolic disease were estimated with the increasing numbers of children across various subgroups (Supplementary Figure 1). Besides education, the difference in probabilities was obvious between subgroups of other confounding factors.

### Unmeasured confounding assessment

As only four confounding factors were adjusted and stratified, the possibility of unmeasured confounding cannot be fully ruled out. To assess this possibility, the E-value was calculated accordingly. As shown in Supplementary Table 6, the E-values were in the range of 1.37–1.43 for three significant exposures—number of children, age at menarche, and history of abortion—which were large compared to the magnitude of corresponding association, meaning that unmeasured confounders, if they exist, must have much larger effects than reproductive factors under investigation to explain away reported association.

## Discussion

The findings of this nationally representative study based on the CHARLS 2014–2018 dataset supported our hypothesis that three reproductive factors—number of children, age at menarche, and history of abortion—were independently and significantly associated with cardiometabolic disease among 6,407 women aged ≥45 years. Moreover, this association was more evident among older women with overweight or obesity. Importantly, sufficient consideration of both measured and unmeasured confounding factors ensures the validity and reliability of these study findings.

It is worth noting that large numbers of children (≥3) were observed as having a greater risk of cardiometabolic disease among middle-aged and older women, and statistical significance persists after statistical adjustment, in line with the results of previous studies (19–21). There is wide recognition that high parity exerts an adverse impact on women's health, as pregnancy usually leads to a series of pathologic and physiological changes in the body. The earliest evidence in favor of parity and cardiovascular disease in women was reported in the early 1990s (22). Later, the number of publications on this aspect increased at a rapid pace, mostly revolving around cardiovascular and metabolic health. For instance, blood lipids and blood pressures go up and down during pregnancy, and these changes usually cause a high incidence of coronary heart disease in later life (23–25). Moreover, insulin tends to increase under the impact of gestational hormones during pregnancy, especially in the case of high parity after two children, which could result in accumulating physiological changes, such as progressive insulin resistance (25). Changes in lipid and insulin profiles were observed to be closely associated with complicated metabolic dysfunction (26), such as abnormal glucose tolerance, dyslipidemia, and obesity (27–29). Hence, each experience of pregnancy means fluctuation of cardiovascular and metabolic profiles. Moreover, some pregnancy complications, such as gestational diabetes, pregnancy hypertension, and pre-eclampsia, were found to be significantly associated with the subsequent development of cardiometabolic disorders (30, 31).

Besides the biological implications, some socioeconomic and lifestyle factors also merit special consideration. There is evidence that mothers with large numbers of children often had an agricultural household, low income, low level of education, and poor medical insurance (5); most of them were more likely to be employed in physical labor or were jobless and malnourished, which can, at least in part, be explained by the obesity-dependent association between number of children and cardiometabolic disease observed in this study. In addition, having more children means more childbearing, more responsibility, more financial stress and parental anxiety, and less sleep, and these unfavorable influences may in turn precipitate the development of cardiovascular and metabolic disorders. Importantly, our findings underscore the need for more attention to the cardiometabolic health of women who have three or more children.

Another important finding of this study is the significant association between age at onset of menarche at 16–18 years and low risk for cardiometabolic disease, consistent with that of previous studies (32, 33). For instance, Qiu et al. reported a significant association between late menarche and decreased cardiovascular disease risk (34). Generally, menarche, the onset of the first menstrual period, is considered the central event of female puberty (35), and age at menarche is complex and under the control of multiple genetic and environmental factors. Interpreting the mechanisms linking age at menarche and cardiometabolic disease is beyond the scope of this study, and we agree that more investigations are warranted.

In addition, we have identified a risk-conferring impact of history of abortion on the odds of cardiometabolic disease. Evidence from a recent meta-analysis showed that women with previous pregnancy loss were at high risk for cardiovascular disease and stroke in later life (36), which consolidated our observations.

### Limitations

Besides the clear strengths of this study, including the first interrogation of reproductive factors and cardiometabolic disease in a nationally representative sample of Chinese middle-aged and older women and careful consideration of potential confounding factors, some limitations should be acknowledged. First, information on cardiovascular and metabolic diseases was gleaned via self-reported questionnaires and recall bias cannot be fully excluded. Second, cardiometabolic disease was determined based on questions regarding the diagnosis of the condition by a doctor, which cannot exclude the possibility of selection bias, as a person who has diabetes has not visited a doctor. Third, although the CHARLS is designed as longitudinal surveys, our analyses were cross-sectional in nature based on data from the latest survey in 2018. Fourth, only four confounders were considered in this study, and the possibility of unknown or residual confounding cannot be excluded. As reflected by unmeasured confounding assessment, if unknown confounding can explain our findings, the odds of its association with cardiometabolic disease must exceed 1.37, which seems unlikely in view of the association magnitude observed in this study. Fifth, all study participants are of Chinese descent, and the extrapolation of our findings to other ethnic groups should be done with caution.

## Conclusion

Taken together, our findings of the national CHARLS database demonstrated that number of children, age at menarche, and history of abortion were independently associated with a significant risk of cardiometabolic disease among 6,407 women aged ≥45 years. Early identification and prevention of cardiometabolic disease based on reproductive factors should be a public health priority to reduce cardiovascular and metabolism-related morbidity and mortality in China.

## Data Availability

The original contributions presented in the study are included in the article/**Supplementary Material**, further inquiries can be directed to the corresponding authors.
