# Supplementary material for: Reproductive factors and cardiometabolic disease among middle-aged and older women: a nationwide study from CHARLS
Source: Front Cardiovasc Med. 2024 Apr 30;11:1345186. doi: 10.3389/fcvm.2024.1345186 (PMC11091256; doi:10.3389/fcvm.2024.1345186)
Supplement: Supplementary file 1 [file Datasheet1.docx]

**Supplementary Table 1.** Overall association of reproductive factors with the risk for hypertension.

| **Reproductive factors** | **Model 1** | | **Model 2** | | **Model 3** | |
| --- | --- | --- | --- | --- | --- | --- |
| Number of children | OR | 95% CI | OR | 95% CI | OR | 95% CI |
| 0-1 | 0.585^***^ | (0.519, 0.659) | 0.938 | (0.804, 1.095) | 0.874 | (0.733, 1.042) |
| 2 | Ref. | | Ref. | | Ref. | |
| ≥3 | 0.875^***^ | (0.815, 0.939) | 1.173^*^ | (1.032, 1.333) | 1.201^*^ | (1.043, 1.382) |
| Age at first livebirth |  |  |  |  |  |  |
| <25 years | 0.581^***^ | (0.484, 0.698) | 0.911 | (0.735, 1.129) | 0.831 | (0.653, 1.057) |
| 25-34 years | Ref. | | Ref. | | Ref. | |
| ≥34 years | 0.658^**^ | (0.496, 0.875) | 0.898 | (0.654, 1.232) | 0.912 | (0.646, 1.287) |
| Age at menarche |  |  |  |  |  |  |
| ≤16 years | Ref. | | Ref. | | Ref. | |
| 16-18 years | 0.720^***^ | (0.657, 0.788) | 0.847^**^ | (0.746, 0.961) | 0.828^**^ | (0.722, 0.950) |
| >18 years | 0.712^***^ | (0.642, 0.790) | 0.784^***^ | (0.683, 0.900) | 0.812^**^ | (0.700, 0.942) |
| Age at menopause |  |  |  |  |  |  |
| ≤45 years | 0.452^***^ | (0.355, 0.576) | 1.001 | (0.701, 1.429) | 0.918 | (0.597, 1.410) |
| 45-55 years | Ref. | | Ref. | | Ref. | |
| ≥55 years | 0.538 | (0.281, 1.031) | 0.897 | (0.433, 1.859) | 0.738 | (0.278, 1.959) |
| Fertile lifespan |  |  |  |  |  |  |
| ≤33 years | 0.473^***^ | (0.371, 0.603) | 0.946 | (0.645, 1.389) | 0.860 | (0.556, 1.328) |
| 33-43 years | Ref. | | Ref. | | Ref. | |
| ≥43 years | NA | NA | NA | NA | NA | NA |
| History of abortion | 0.696^***^ | (0.595, 0.814) | 1.047 | (0.883, 1.242) | 1.081 | (0.894, 1.307) |

Abbreviations: Ref., reference group; NA, data not available; OR, odds ratio; 95% CI, 95% confidence interval.

**p*<0.05, ***p*<0.01, ****p*<0.001.

Model 1: no confounder was adjusted.

Model 2: age (continuous), residential area (rural, urban), and education (illiterate, literate) were adjusted.

Model 3: age (continuous), residential area (rural, urban), education (illiterate, literate), and body mass index were adjusted.

**Supplementary Table 2.** Overall association of reproductive factors with the risk for dyslipidemia.

| **Reproductive factors** | **Model 1** | | **Model 2** | | **Model 3** | |
| --- | --- | --- | --- | --- | --- | --- |
| Number of children | OR | 95% CI | OR | 95% CI | OR | 95% CI |
| 0-1 | 0.306^***^ | (0.267, 0.351) | 0.800^*^ | (0.668, 0.958) | 0.763^*^ | (0.621, 0.938) |
| 2 | Ref. | | Ref. | | Ref. | |
| ≥3 | 0.268^***^ | (0.245, 0.293) | 1.020 | (0.877, 1.187) | 1.029 | (0.873, 1.212) |
| Age at first livebirth |  |  |  |  |  |  |
| <25 years | 0.261^***^ | (0.209, 0.325) | 0.790 | (0.612, 1.019) | 0.732^*^ | (0.550, 0.973) |
| 25-34 years | Ref. | | Ref. | | Ref. | |
| ≥34 years | 0.236^***^ | (0.165, 0.337) | 0.851 | (0.576, 1.257) | 0.913 | (0.598, 1.394) |
| Age at menarche |  |  |  |  |  |  |
| ≤16 years | Ref. | | Ref. | | Ref. | |
| 16-18 years | 0.276^***^ | (0.247, 0.308) | 0.862 | (0.741, 1.002) | 0.865 | (0.735, 1.018) |
| >18 years | 0.278^***^ | (0.245, 0.315) | 0.931 | (0.789, 1.099) | 0.988 | (0.827, 1.180) |
| Age at menopause |  |  |  |  |  |  |
| ≤45 years | 0.279^***^ | (0.213, 0.366) | 1.137 | (0.755, 1.712) | 0.896 | (0.550, 1.460) |
| 45-55 years | Ref. | | Ref. | | Ref. | |
| ≥55 years | 0.0789^***^ | (0.0244, 0.256) | 0.369 | (0.107, 1.264) | 0.183 | (0.0233, 1.435) |
| Fertile lifespan |  |  |  |  |  |  |
| ≤33 years | 0.280^***^ | (0.213, 0.369) | 1.085 | (0.695, 1.695) | 1.083 | (0.654, 1.795) |
| 33-43 years | Ref. | | Ref. | | Ref. | |
| ≥43 years | NA | NA | NA | NA | NA | NA |
| History of abortion | 0.404^***^ | (0.340, 0.480) | 1.285^*^ | (1.061, 1.555) | 1.301^*^ | (1.051, 1.609) |

Abbreviations: Ref., reference group; NA, data not available; OR, odds ratio; 95% CI, 95% confidence interval.

**p*<0.05, ***p*<0.01, ****p*<0.001.

Model 1: no confounder was adjusted.

Model 2: age (continuous), residential area (rural, urban), and education (illiterate, literate) were adjusted.

Model 3: age (continuous), residential area (rural, urban), education (illiterate, literate), and body mass index were adjusted.

**Supplementary Table 3.** Overall association of reproductive factors with the risk for diabetes mellitus.

| **Reproductive factors** | **Model 1** | | **Model 2** | | **Model 3** | |
| --- | --- | --- | --- | --- | --- | --- |
| Number of children | OR | 95% CI | OR | 95% CI | OR | 95% CI |
| 0-1 | 0.142^***^ | (0.119, 0.169) | 0.858 | (0.683, 1.078) | 0.816 | (0.631, 1.054) |
| 2 | Ref. | | Ref. | | Ref. | |
| ≥3 | 0.163^***^ | (0.147, 0.181) | 1.237^*^ | (1.028, 1.489) | 1.249^*^ | (1.025, 1.522) |
| Age at first livebirth |  |  |  |  |  |  |
| <25 years | 0.148^***^ | (0.113, 0.193) | 0.888 | (0.650, 1.213) | 0.791 | (0.559, 1.121) |
| 25-34 years | Ref. | | Ref. | | Ref. | |
| ≥34 years | 0.200^***^ | (0.138, 0.291) | 1.406 | (0.924, 2.141) | 1.384 | (0.882, 2.172) |
| Age at menarche |  |  |  |  |  |  |
| ≤16 years | Ref. | | Ref. | | Ref. | |
| 16-18 years | 0.159^***^ | (0.139, 0.181) | 1.001 | (0.833, 1.202) | 0.985 | (0.810, 1.198) |
| >18 years | 0.156^***^ | (0.134, 0.182) | 1.004 | (0.820, 1.228) | 1.016 | (0.821, 1.259) |
| Age at menopause |  |  |  |  |  |  |
| ≤45 years | 0.114^***^ | (0.0783, 0.165) | 0.946 | (0.553, 1.618) | 0.708 | (0.372, 1.347) |
| 45-55 years | Ref. | | Ref. | | Ref. | |
| ≥55 years | 0.0789^***^ | (0.0244, 0.256) | 0.649 | (0.182, 2.315) | 0.560 | (0.119, 2.631) |
| Fertile lifespan |  |  |  |  |  |  |
| ≤33 years | 0.113^***^ | (0.0773, 0.165) | 0.971 | (0.540, 1.746) | 0.833 | (0.437, 1.588) |
| 33-43 years | Ref. | | Ref. | | Ref. | |
| ≥43 years | NA | (0.0625, 15.99) | 5.496 | (0.271, 111.4) | NA | NA |
| History of abortion | 0.156^***^ | (0.124, 0.196) | 0.957 | (0.747, 1.225) | 0.977 | (0.748, 1.276) |

Abbreviations: Ref., reference group; NA, data not available; OR, odds ratio; 95% CI, 95% confidence interval.

**p*<0.05, ***p*<0.01, ****p*<0.001.

Model 1: no confounder was adjusted.

Model 2: age (continuous), residential area (rural, urban), and education (illiterate, literate) were adjusted.

Model 3: age (continuous), residential area (rural, urban), education (illiterate, literate), and body mass index were adjusted.

**Supplementary Table 4.** Overall association of reproductive factors with the risk for heart problems.

| **Reproductive factors** | **Model 1** | | **Model 2** | | **Model 3** | |
| --- | --- | --- | --- | --- | --- | --- |
| Number of children | OR | 95% CI | OR | 95% CI | OR | 95% CI |
| 0-1 | 0.285^***^ | (0.248, 0.328) | 0.781^**^ | (0.653, 0.935) | 0.745^**^ | (0.605, 0.919) |
| 2 | Ref. | | Ref. | | Ref. | |
| ≥3 | 0.382^***^ | (0.353, 0.414) | 1.315^***^ | (1.138, 1.520) | 1.308^**^ | (1.114, 1.535) |
| Age at first livebirth |  |  |  |  |  |  |
| <25 years | 0.250^***^ | (0.200, 0.312) | 0.713^**^ | (0.553, 0.920) | 0.677^**^ | (0.508, 0.903) |
| 25-34 years | Ref. | | Ref. | | Ref. | |
| ≥34 years | 0.256^***^ | (0.181, 0.363) | 0.780 | (0.534, 1.141) | 0.737 | (0.487, 1.115) |
| Age at menarche |  |  |  |  |  |  |
| ≤16 years | Ref. | | Ref. | | Ref. | |
| 16-18 years | 0.343^***^ | (0.309, 0.380) | 0.888 | (0.770, 1.023) | 0.894 | (0.765, 1.044) |
| >18 years | 0.316^***^ | (0.280, 0.357) | 0.828^*^ | (0.707, 0.970) | 0.827^*^ | (0.697, 0.982) |
| Age at menopause |  |  |  |  |  |  |
| ≤45 years | 0.169^***^ | (0.122, 0.232) | 0.970 | (0.610, 1.545) | 0.950 | (0.541, 1.669) |
| 45-55 years | Ref. | | Ref. | | Ref. | |
| ≥55 years | 0.355^**^ | (0.178, 0.706) | 2.035 | (0.916, 4.520) | 1.386 | (0.461, 4.173) |
| Fertile lifespan |  |  |  |  |  |  |
| ≤33 years | 0.190^***^ | (0.139, 0.259) | 1.122 | (0.680, 1.850) | 1.250 | (0.698, 2.239) |
| 33-43 years | Ref. | | Ref. | | Ref. | |
| ≥43 years | NA | (0.0625, 15.99) | 4.173 | (0.230, 75.82) | NA | NA |
| History of abortion | 0.369^***^ | (0.310, 0.439) | 1.033 | (0.854, 1.250) | 1.087 | (0.879, 1.345) |

Abbreviations: Ref., reference group; NA, data not available; OR, odds ratio; 95% CI, 95% confidence interval.

**p*<0.05, ***p*<0.01, ****p*<0.001.

Model 1: no confounder was adjusted.

Model 2: age (continuous), residential area (rural, urban), and education (illiterate, literate) were adjusted.

Model 3: age (continuous), residential area (rural, urban), education (illiterate, literate), and body mass index were adjusted.

**Supplementary Table 5.** Overall association of reproductive factors with the risk for stroke.

| **Reproductive factors** | **Model 1** | | **Model 2** | | **Model 3** | |
| --- | --- | --- | --- | --- | --- | --- |
| Number of children | OR | 95% CI | OR | 95% CI | OR | 95% CI |
| 0-1 | 0.0646^***^ | (0.0507, 0.0822) | 0.732^*^ | (0.544, 0.983) | 0.779 | (0.556, 1.092) |
| 2 | Ref. | | Ref. | | Ref. | |
| ≥3 | 0.109^***^ | (0.0962, 0.122) | 1.235 | (0.992, 1.537) | 1.194 | (0.935, 1.526) |
| Age at first livebirth |  |  |  |  |  |  |
| <25 years | 0.0846^***^ | (0.0610, 0.117) | 1.022 | (0.693, 1.506) | 1.067 | (0.695, 1.638) |
| 25-34 years | Ref. | | Ref. | | Ref. | |
| ≥34 years | 0.0737^***^ | (0.0428, 0.127) | 0.925 | (0.508, 1.686) | 0.810 | (0.413, 1.589) |
| Age at menarche |  |  |  |  |  |  |
| ≤16 years | Ref. | | Ref. | | Ref. | |
| 16-18 years | 0.0887^***^ | (0.0752, 0.105) | 0.857 | (0.686, 1.071) | 0.935 | (0.733, 1.194) |
| >18 years | 0.0969^***^ | (0.0810, 0.116) | 0.925 | (0.728, 1.175) | 0.990 | (0.763, 1.283) |
| Age at menopause |  |  |  |  |  |  |
| ≤45 years | 0.0614^***^ | (0.0382, 0.0989) | 0.910 | (0.458, 1.809) | 0.932 | (0.394, 2.205) |
| 45-55 years | Ref. | | Ref. | | Ref. | |
| ≥55 years | 0.0789^***^ | (0.0244, 0.256) | 1.099 | (0.294, 4.108) | 0.647 | (0.0747, 5.601) |
| Fertile lifespan |  |  |  |  |  |  |
| ≤33 years | 0.0596^***^ | (0.0366, 0.0973) | 0.886 | (0.418, 1.881) | 0.956 | (0.403, 2.269) |
| 33-43 years | Ref. | | Ref. | | Ref. | |
| ≥43 years | NA | NA | NA | NA | NA | NA |
| History of abortion | 0.0955^***^ | (0.0728, 0.125) | 1.092 | (0.813, 1.466) | 1.106 | (0.795, 1.538) |

Abbreviations: Ref., reference group; NA, data not available; OR, odds ratio; 95% CI, 95% confidence interval.

**p*<0.05, ***p*<0.01, ****p*<0.001.

Model 1: no confounder was adjusted.

Model 2: age (continuous), residential area (rural, urban), and education (illiterate, literate) were adjusted.

Model 3: age (continuous), residential area (rural, urban), education (illiterate, literate), and body mass index were adjusted.

**Supplementary Table 6.** The E-values of reproductive factors with the risk of cardiometabolic disease.

| **Number of children** | |  | **Age at menarche** | |  | **History of abortion** | |
| --- | --- | --- | --- | --- | --- | --- | --- |
| 2 | Ref. |  | ≤16 years | Ref. |  | No | Ref. |
| 0-1 | 1.40 (1.03) |  | 16-18 years | 1.37 (1.11) |  | Yes | 1.43 (1.06) |
| ≥3 | 1.39 (1.13) |  |  |  |  |  |  |

The values in parentheses denotes the lower limits of E-values. Abbreviations: Ref., reference group.

**Supplementary Figure 1.** Identification of optimal age threshold in predicting the risk for cardiometabolic disease using restricted cubic spline analysis.


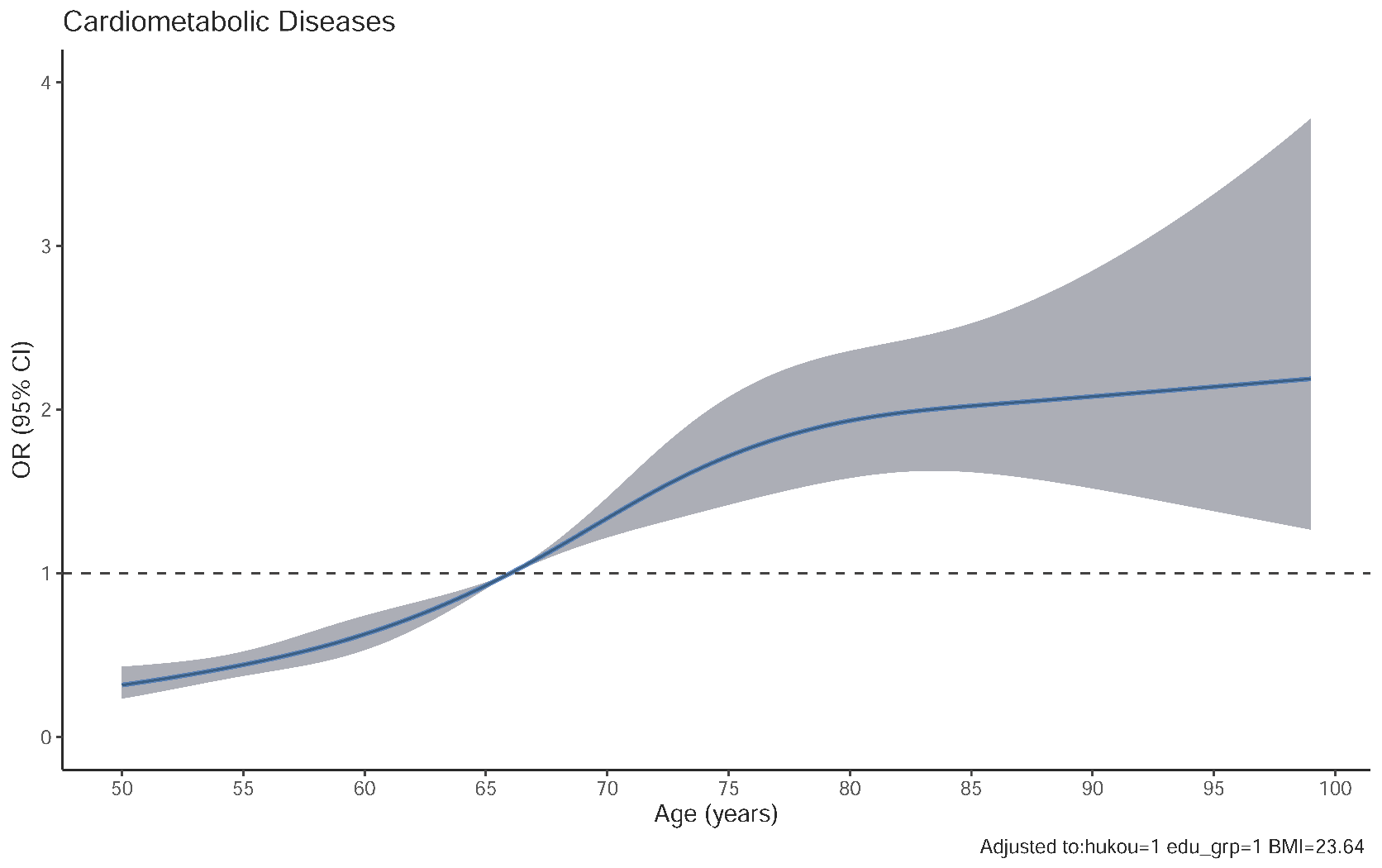


Abbreviations: OR, odds ratio; 95% CI, 95% confidence interval.
